# Sub-analysis of geographical variations in the 2-year observational COPTIMIZE trial of patients with relapsing–remitting multiple sclerosis converting to glatiramer acetate

**DOI:** 10.1186/s12883-015-0448-4

**Published:** 2015-10-08

**Authors:** Tjalf Ziemssen, Yossi Gilgun-Sherki

**Affiliations:** Center of Clinical Neuroscience, Neurological University Clinic, University Clinic Carl Gustav Carus, University of Technology Dresden, Fetscherstraße 74, D-01307 Dresden, Germany; Teva Pharmaceutical Industries Ltd, 5 Basel Street, Petah Tikva, 49131 Israel

**Keywords:** Multiple sclerosis, RRMS, Glatiramer acetate, Demographics

## Abstract

**Background:**

Studies suggest that patients with relapsing–remitting multiple sclerosis (RRMS) who fail to benefit from a disease-modifying treatment (DMT) may benefit from converting to another DMT class. COPTIMIZE was a 24-month observational study designed to assess the disease course of patients converting to glatiramer acetate (GA) 20 mg daily from another DMT and the association of disease characteristics and reasons for converting. This sub-analysis was to determine if any findings varied by three geographic locations: Latin America (LA), Canada and Western Europe (CWE), and Eastern Europe (EE).

**Methods:**

A total of 668 patients were included (263 LA, 248 CWE, 157 EE) in an analysis of annualized relapse rate (ARR) and annualized rate of deterioration (ARD), as well as secondary endpoints including reason for DMT switch and changes in disability and fatigue scores. Repeated-measures analysis of variance and log transformation were used to analyze ARR and ARD, whereas the Wilcoxon signed rank test was used for secondary endpoints.

**Results:**

The sub-analysis of treatment outcomes stratified by region showed that Latin American patients had higher ARR before conversion to GA compared with patients from the other two areas and subsequently experienced the largest reduction in ARR. Latin American patients also had higher baseline rates of comorbidities and relapses with incomplete remissions and improved more than those in the other two regions based on measures of fatigue, quality of life, depression, and cognition scores. Latin American patients also generally had a better perception of the benefits associated with their conversion to GA in terms of efficacy and adverse events.

**Conclusions:**

These findings indicate that, in RRMS patients, converting to GA is associated with positive treatment outcomes regardless of geographic location. However, the reasons for converting and the type and degree of any associated benefits appear to vary depending on various factors, including patients’ geographical location.

**Electronic supplementary material:**

The online version of this article (doi:10.1186/s12883-015-0448-4) contains supplementary material, which is available to authorized users.

## Background

Multiple sclerosis (MS) is a chronic relapsing disorder of the central nervous system characterized by inflammation, multifocal demyelination, and neuronal and axonal damage [1]. The majority of MS patients initially present with relapsing–remitting MS (RRMS) that frequently develops to a progressive disease course [1]. The prevalence of MS varies according to geographic location from 10 to 20 per 100,000 in Central and South America to >30 per 100,000 in northern Europe and North America [2].

Immunomodulating disease-modifying therapies (DMTs) have been shown to improve multiple measures of disease activity in RRMS patients, including the annualized relapse rate (ARR), proportion of relapse-free individuals, and accumulation of T2 lesion burden [3–5]. However, these agents are only partially effective in controlling disease progression; studies have reported treatment interruption or discontinuation because of lack of tolerability, progression of disability, or inadequate clinical response [6]. Additionally, the development of neutralizing antibodies, specifically with interferon β products (IFNs) and natalizumab [7], can interfere with the biologic response [8].

Converting to another DMT class represents one treatment strategy for MS patients with an inadequate response to first-line treatments or intolerant side effects [9]. Expert guidance on the specific steps of a conversion has been reported [10]; however, the lack of information on outcomes in different populations [11] results in limited guidance on regional patient considerations.

The COPTIMIZE study was designed to monitor clinical outcomes after converting from failing or ineffective DMT therapy for RRMS to glatiramer acetate (GA) in a prospective way. GA is approved in 57 countries as a 20 mg daily subcutaneous (s.c.) injection for reducing relapse frequency in patients with RRMS. It has long-term efficacy and safety data, with the longest continuous treatment exposure of more than 20 years [12, 13]. The primary results of COPTIMIZE, presented elsewhere [14], indicate that a conversion to GA is associated with positive treatment outcomes and that the benefits vary depending on patients’ reasons for changing. The primary objective of the present sub-analysis is to determine whether benefits associated with converting to GA were affected by therapeutic strategies and patient selection in different geographic locations: Latin America (LA), Canada and Western Europe (CWE), and Eastern Europe (EE).

## Methods

### Study design

Study design, patient eligibility criteria, and conduct of the COPTIMIZE have been previously reported [14]. Briefly, this post-hoc subgroup analysis attempted to describe any variation in results that might exist between three predefined geographical areas: LA (Argentina, Brazil, Chile, Mexico, Venezuela), CWE (Belgium, Canada, Denmark, France, Greece, Ireland, Portugal, Netherlands, Norway, Sweden), and EE (Hungary, Romania, Slovakia). Countries were grouped into regions based on similarity of healthcare systems, physician approaches [15], available treatment options [10, 16], and epidemiological characteristics of the population (disease prevalence, demographics, etc.) [2]. All countries investigated in this observational study reported the use of IFN-β and GA at baseline, with no anticipated systematic differences between regions. This study was conducted in accordance with the 18th World Medical Assembly (Helsinki) recommendations and amendments, as well as guidelines for Good Epidemiology Practice. The study protocol was approved by the institutional review boards and independent ethics committees at all participating study locations in each individual country; each site ensured all necessary regulatory submissions in accordance with local regulations including local data protection regulations. All patients provided informed, written consent according to local independent review board ruling.

### Study endpoints

The primary objective was to assess the disease course in RRMS patients converting from IFN treatment to GA as measured by the primary endpoints of ARR and annualized rate of deterioration (ARD) (confirmed progression of Expanded Disability Status Scale [EDSS]/worsening mobility scores). Secondary data collected included reasons for DMT conversion, characteristics of patients failing to benefit from previous DMT, and change in EDSS and modified fatigue impact scale (MFIS) scores. Also recorded were quality of life (QoL) changes following GA conversion as measured by the Functional Assessment of Multiple Sclerosis (FAMS), cognition changes as evaluated by the Paced Auditory Serial Addition Test (PASAT), depression as measured by Centre for Epidemiologic Studies Depression (CES-D) scale, and change in adverse events (AEs) following the conversion.

### Statistical analyses

Statistical analyses of parameters in this observational study required comparison of at least two endpoint measures, pre- and post-GA conversion, with data represented by descriptive procedures and figures, if necessary. Adjustment for missing data was not required to maintain statistical integrity of the analyses, and annualized rates (primary endpoints) were calculated for each subject using all the available data. Other parameters, which provide additional data for evaluation of the patient status prior to and following conversion to GA, were reported in a non-obligatory manner. Tests of significance (signal rank test and binominal test) were used to measure changes in efficacy parameters from baseline to final examination. ARR and ARD pre- and post-conversion were analyzed using repeated measures analysis of covariance using the maximum likelihood ratio. Log transformation was implemented to the ARR and ARD to establish if there was a significant deviation from normality (i.e., *P* < .001 using the Shapiro-Wilk test). The Wilcoxon signed rank test was used within groups for EDSS, MFIS, FAMS, PASAT, and CES-D.

## Results

### Patient disposition

Overall, 672 patients from 148 centers across 19 countries were enrolled, with 668 patients included in the analysis (excluding four patients from Taiwan): 263 LA, 248 CWE, and 157 EE patients (Table 1). Patient characteristics were comparable at baseline between regions, excluding EE patients, who were younger and reported fewer comorbidities and concomitant medications than LA and CWE patients. Baseline disease characteristics that were similar across regions (Table 1) included disease duration from onset, diagnosis, and mean ARR. Distribution of baseline ARR score varied slightly, with the majority of patients experiencing between 0 and 1.25 events/year. LA patients reported the highest disability (baseline EDSS) and the highest frequency of RRMS with incomplete remissions. At baseline, the majority of patients had received one previous DMT regimen in one class of agents (Table 1). Reports of flu-like symptoms were the most common reason for converting to GA. The majority of patients in all regions were converted from IFN-β therapy.

**Table 1:** Baseline patient demographics, disease characteristics, and DMT history

Characteristics	LA (*n* = 263)	CWE (*n* = 248)	EE (*n* = 157)
Female gender, *n* (%)	189 (71.9)	175 (70.6)	108 (68.8)
Mean age, years (± SD)	40.1 (10.1)	43.0 (10.2)	34.7 (8.4)
Patients with comorbidities at recruitment, *n* (%)	27 (10.3)	19 (7.7)	8 (5.1)
Depression	13 (4.9)	6 (2.4)	1 (0.6)
Anxiety	2 (0.8)	1 (0.4)	1 (0.6)
Hypertension	1 (0.4)	4 (1.6)	2 (1.3)
Patients with concomitant therapies at time of recruitment, *n* (%)	24 (9.1)	15 (6.1)	7 (4.5)
Psychoanaleptics	14 (5.3)	5 (2.0)	1 (0.6)
Antiepileptics	6 (2.3)	2 (0.8)	1 (0.6)
Thyroid therapy	3 (1.1)	3 (1.2)	N/A
Mean disease duration since onset, months (± SD)^a^	98.0 (82.9)	100.1 (84.4)	92.3 (63.9)
Mean disease duration since diagnosis, months (± SD)^b^	68.9 (59.6)	72.1 (70.7)	67.9 (48.5)
Mean ARR, events/year (± SD)^c^	1.0 (0.8)	0.8 (0.6)	0.7 (0.5)
Patients in ARR range, *n* (%*)			
0.00–1.25	166 (67.5)	193 (84.6)	130 (88.4)
1.25–3.25	78 (31.7)	35 (15.4)	17 (11.6)
>3.25	2 (0.8)	0 (0.0)	0 (0.0)
Data unavailable	17	20	10
Clinical type of MS, *n* (%^d^)			
RRMS with incomplete remissions	171 (67.6)	117 (47.6)	91 (59.1)
RRMS with complete remission	80 (31.6)	122 (49.6)	62 (40.3)
Clinically isolated syndrome	0 (0.0)	0 (0.0)	1 (0.6)
Other	2 (0.8)	7 (2.8)	0 (0.0)
Data unavailable	10	2	3
Diagnosed with MS by criteria, *n* (%^d^)			
McDonald	217 (83.8)	194 (78.5)	143 (92.3)
Poser	42 (16.2)	53 (21.5)	12 (7.7)
Data unavailable	4	1	2
Mobility, *n* (%^d^)			
Asymptomatic	45 (19.8)	46 (20.0)	20 (14.9)
Able to walk unaided for >500 m	96 (42.3)	126 (54.8)	112 (83.6)
Able to walk unaided for <500 m	30 (13.2)	27 (11.7)	2 (1.5)
Walking with bilateral support	13 (5.7)	9 (3.9)	0 (0.0)
Walking with unilateral support	33 (14.5)	18 (7.8)	0 (0.0)
Need of a wheelchair outdoors	10 (4.4)	4 (1.7)	0 (0.0)
Data unavailable	36	18	23
Mean EDSS score (± SD)^e^	3.5 (2.2)	2.8 (1.9)	2.6 (1.0)
Mean CES-D score (0–60) (± SD)^f^	16.0 (11.7)	16.0 (10.3)	20.6 (19.5)
Mean MFIS score (0–84) (± SD)^g^	32.3 (19.7)	31.4 (19.1)	33.7 (27.9)
Mean FAMS score (0–176) (± SD)^h^	109.4 (37.8)	100.8 (34.3)	77.7 (59.4)
Mean PASAT score (0–60) (± SD)^i^	35.6 (13.6)	36.7 (15.7)	51.8 (5.9)
Mean observation duration, months (± SD)^j^	20.5 (6.3)	18.6 (7.7)	19.2 (7.8)
Number of DMT classes used (%) (converters only)			
1	206 (85.5)	201 (83.4)	143 (92.9)
2	32 (13.3)	38 (15.8)	10 (6.5)
3	3 (1.2)	2 (0.8)	1 (0.6)
Non-converters	22	7	3
Previous type and mode of IFN-β used, %^k^			
IFN-β-1a (i.m.)	30.3	36.0	47.4
IFN-β-1b (s.c.)	30.3	25.9	30.5
IFN-β-1a (s.c.)	35.5	30.7	21.4
Reason for conversion to GA, *n* (%)^l^			
Lack of previous DMT efficacy	171 (71.0)	78 (32.4)	92 (59.7)
Presence of neutralizing antibodies	1 (0.4)	44 (18.3)	2 (1.3)
Intolerable adverse events associated with previous DMT	98 (40.7)	132 (54.8)	55 (35.7)
Flu–like symptoms	67 (27.8)	73 (30.3)	40 (26.0)
Subjective	29 (12.0)	37 (15.4)	17 (11.0)
Skin reactions	15 (6.2)	14 (5.8)	20 (13.0)
Blood work	7 (2.9)	18 (7.5)	4 (2.6)
Others	21 (8.7)	39 (16.2)	4 (2.6)
Not specified	1 (0.4)	1 (0.4)	0 (0.0)
Other	8 (3.3)	21 (8.7)	36 (23.4)
Non-converters	22	7	3
Discontinuation of GA, *n* (%)	66 (25.1)	77 (31.1)	30 (19.1)
Perceived lack of efficacy by physician	19 (7.2)	7 (2.8)	16 (10.2)
Perceived lack of efficacy by patient	7 (2.7)	14 (5.7)	6 (3.8)
Adverse events	9 (3.4)	17 (6.9)	4 (2.5)
Lost to follow-up	14 (5.3)	24 (9.7)	2 (1.3)
Other	15 (5.7)	8 (3.2)	3 (1.9)

^a^Missing data in 24 LA, 28 CWE, and 5 EE patients

^b^Missing data in 15 LA, 21 CWE, and 4 EE patients

^c^Missing data in 7 LA, 2 CWE, and 3 EE patients

^d^Adjusted percentage of patients with data available

^e^Missing data in 55 LA, 9 CWE, and 8 EE patients

^f^Missing data in 116 LA, 103 CWE, and 150 EE patients

^g^Missing data in 113 LA, 118 CWE, and 150 EE patients

^h^Missing data in 55 LA, 9 CWE, and 8 EE patients

^i^Missing data in 197 LA, 103 CWE, and 150 EE patients

^j^Missing data in 11 LA, 17 CWE, and 10 EE patients

^k^Missing data in 32 LA, 20 CWE, and 3 EE patients

^l^Adjusted percentage of patients with data available. Patients were allowed to cite ≥1 reason for conversion to GA. Therefore, the percentage may exceed 100 %

### Annualized relapse rate

**Fig. 1:**
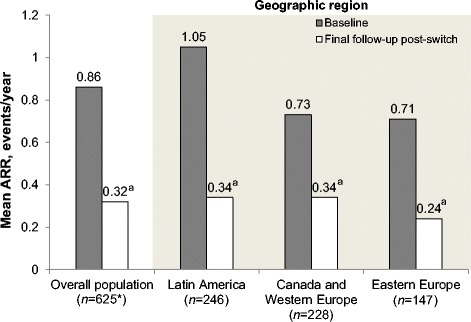
Reduction of annualized relapse rate by geographic region. ^a^ *P* < .0001 vs. baseline. *The overall population size does not each the sum of the three individual regions due to four patients in Taiwan that excluded from the regional analysis. *ARR* annualized relapse rate

ARR was significantly decreased in all groups following the conversion to GA (Fig. 1). LA patients, who had the highest baseline ARR rate, also had the greatest ARR reduction (1.05 ± 0.78 pre-conversion to 0.34 ± 0.86 post-conversion; *P* < .0001, Shapiro-Wilk test). ARR went from 0.73 ± 0.58 to 0.34 ± 0.84 (*P* < .0001) in CWE patients and 0.71 ± 0.50 to 0.24 ± 0.92 (*P* < .0001) in EE patients.

#### Disease progression

In total, 499 patients had ≥1 EDSS assessment after baseline examination, with the overall population showing a significant increase in EDSS score (i.e., progression to worse disability) from 2.9 at baseline to 3.02 at final follow-up, post-switch (*P* = .0256). There was a significant difference between regions in the degree of change in EDSS score while on GA therapy (*P* = .0230, Wilcoxon signed-rank test), driven by a significant increase in CWE patients’ EDSS score of 0.26 ± 1.18 (*P* = .0016). Neither LA nor EE patients had significant changes in mean EDSS score from baseline. Improved (i.e., numerically lowered) EDSS scores were seen in 32.5 %, 24.4 %, and 33.8 % of LA, CWE, and EE patients, respectively. There was no change from baseline in 35.8 %, 34.9 %, and 41.2 %, respectively. Deterioration was reported in 31.8 %, 40.7 %, and 25.0 %, respectively.

#### Disease activity

Disease activity while receiving GA varied. LA patients reported the highest incidence of frequent exacerbations and of fast progression of MS (Table 2). Fewer LA patients reported rarely experiencing exacerbations (27.9 %, vs. 43.5 % CWE and 66.0 % EE patients).

**Table 2:** MS disease activity over the 2-year study period

Patients, *n* (%)	LA	CWE	EE
	(*n* = 251)	(*n* = 246)	(*n* = 153)
Stable MS (Stage 1)	37 (14.7)	47 (19.1)	22 (14.4)
Rare exacerbations (≤1 per year, Stage 2a)	70 (27.9)	107 (43.5)	101 (66.0)
Slow progression (≤0.5 EDSS points per year, Stage 2b)	48 (19.1)	35 (14.2)	6 (3.9)
Frequent exacerbations (>1 per year, Stage 3a)	80 (31.9)	43 (17.5)	21 (13.7)
Fast progression (>0.5 EDSS points per year, Stage 3b)	11 (4.4)	3 (1.2)	1 (0.7)
Not classified/not available	5 (2.0)	11 (4.5)	2 (1.3)

#### Change in mobility

The majority of patients showed no change in mobility scores (63.4 %, 62.8 %, and 67.5 % of LA, CWE, and EE patients, respectively). Mobility scores improved in 17.1 % LA and 18.9 % CWE patients, with a significant improvement in 23.0 % of EE patients (*P* = .0079). Mobility scores deteriorated in 19.4 %, 18.4 %, and 9.5 % of LA, CWE, and EE patients, respectively. Data were missing for 47 LA, 52 CWE, and 31 EE patients.

### Secondary endpoints

**Fig. 2:**
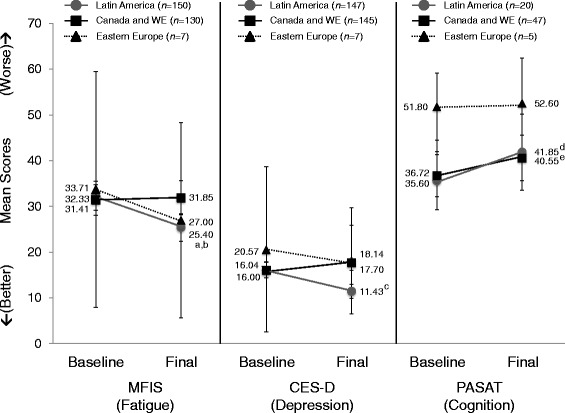
Change in fatigue, depression, and cognition scores by geographic region. ^a^ *P* < .0001 for Latin America vs. Canada/Western Europe at final follow-up. ^b^ *P* < .0001 for change from baseline. ^*c*^ *P* < .0001 for change from baseline. ^*d*^ *P* = .0030 for change from baseline. ^e^ *P* = .0024 for change from baseline. *CES-D* Centre for Epidemiologic Studies Depression, *MFIS* Modified Fatigue Impact Scale, *PASAT* Paced Auditory Serial Addition Test, *WE* Western Europe

In LA patients, there was a significant improvement in the mean difference in MFIS score from baseline (6.9 ± 15.4; *P* < .0001) (Fig. 2). EE patients and CWE patients experienced a nonsignificant increase from baseline of 6.7 ± 18.2 and 0.4 ± 13.6, respectively.

**Fig. 3:**
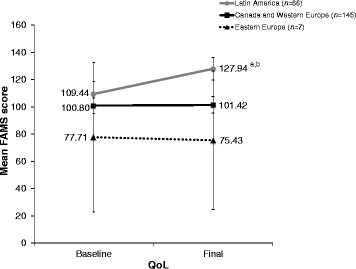
Change in quality of life score by geographic region. ^a^ *P* = .0012 for Latin America vs. Canada/Western Europe at final follow-up. ^b^ *P* = .0008 for change from baseline. *FAMS* Functional Assessment of Multiple Sclerosis

Only LA patients showed a significant change in QoL, with an improvement in mean FAMS score of 18.5 ± 46.5 from baseline (*P* = .0008). CWE patients had a mean FAMS increase of 0.6 ± 20.5, while EE patients showed a decrease of 2.3 ± 8.8 (Fig. 3).

LA patients reported a significant improvement in depression symptoms, with a decrease in mean CES-D score of 4.6 ± 10.9 (*P* < .0001) (Fig. 2). CWE patients showed a numerical increase in mean CES-D score of 1.7 ± 9.9, while EE patients showed a decrease of 2.4 ± 10.8. These changes were not statistically significant.

Patients from all regions showed an improvement in mean PASAT (cognition) scores (Fig. 2). LA patients had the greatest improvement, with a statistically significant mean increase of 6.3 ± 9.1 in PASAT score (*P* = .0030). CWE patients also showed a statistically significant mean increase of 3.8 ± 9.6 (*P* = .0024), while EE patients had a smaller mean increase of 0.8 ± 5.3 (NS).

LA patients reported the highest efficacy with GA than the previous drug (70.0 %; *P* < .0001), followed by EE and CWE patients (42.4 % and 41.6 %, respectively; both *P* < .0001). LA patients reported the lowest percentage of “feeling worse” on GA therapy (3.9 %), with EE patients reporting the highest percentage (15.2 %), followed by CWE patients (6.1 %). There was no change in perception of efficacy in 26.2 %, 52.2 % and 42.4 % of LA, CWE, and EE patients, respectively (Additional file 1).

### Safety

AEs reported in all three regions are listed in Table 3. Patients from all regions experienced statistically significant improvements in AEs with GA use (*P* < .0001).

**Table 3:** Adverse events (AEs)

Patients, events/patients (%^a^)	LA (*n* = 263)	CWE (*n* = 248)	EE (*n* = 157)
Most common AEs by preferred term			
Dyspnea	5/4 (1.5)	4/3 (1.2)	1/1 (0.6)
Syncope	2/2 (0.8)	2/2 (0.8)	0/0 (0.0)
Injection site reaction	2/2 (0.8)	8/8 (3.2)	0/0 (0.0)
Injection site pain	2/2 (0.8)	14/10 (4.0)	0/0 (0.0)
Injection site induration	1/1 (0.4)	5/5 (2.0)	0/0 (0.0)
Fatigue	1/1 (0.4)	3/3 (1.2)	0/0 (0.0)
Arthralgia	1/1 (0.4)	3/3 (1.2)	0/0 (0.0)
Rash	0/0 (0.0)	2/2 (0.8)	2/2 (1.3)
Anxiety	0/0 (0.0)	1/1 (0.4)	1/1 (0.6)
Most common AE classified by system organ class			
General disorders and administration site conditions	16/13 (4.9)	55/32 (12.9)	5/4 (2.6)
Nervous system disorders	10/10 (3.8)	9/8 (3.2)	0/0 (0.0)
Respiratory, thoracic, and mediastinal disorders	7/6 (2.3)	4/3 (1.2)	0/0 (0.0)
Severity of AE, events/patients			
Mild	19/12 (4.6)	40/28 (11.3)	0/0 (0.0)
Moderate	22/12 (4.6)	62/38 (15.3)	5/5 (3.2)
Severe	13/6 (2.3)	14/9 (3.6)	5/3 (1.9)
Data unavailable	4/2 (0.8)	7/5 (2.0)	2/2 (1.3)
Patient-reported assessment of AEs after glatiramer acetate treatment^b^			
Improved with glatiramer acetate	209 (80.4)	162 (66.1)	58 (38.4)
Same with glatiramer acetate	41 (15.8)	62 (25.3)	87 (57.6)
Worse with glatiramer acetate	10 (3.9)	21 (8.6)	6 (4.0)
Data unavailable	3	3	6

^a^Percentage reported as the proportion of patients experiencing events

^b^Adjusted percentage of patients with data available

## Discussion

Converting to another class of immunomodulatory therapy represents one treatment strategy in MS patients who fail to respond adequately to first-line treatments [10]. However, this strategy may not always be beneficial because of geographical variations in treatment regimens and therapeutic strategies. For example, GA and IFN-β are typically the first-line treatment options in MS treatment algorithms, regardless of the geographical region [11]. However, some LA neurologists prescribe azathioprine because of limited DMT access or because the drugs are unavailable on healthcare plans [16, 17]. Expert guidance on the specific steps of a conversion have begun to be published, suggesting a conversion in therapy may be considered when there is a high level of concern about relapse rates, progression of MS and magnetic resonance imaging outcomes, a medium level of concern about any two factors, or a low level of concern about all three factors [10]. The general nature of the guidelines is due to inconsistent results with converting.

In this sub-analysis of the COPTIMIZE trial, converting to GA was well tolerated, reduced disease progression and activity, and improved other secondary endpoints in patients across all three regions to varying degrees. LA patients experienced the largest reduction in ARR; however, their baseline ARR was much higher than CWE and EE patients. Ultimately, all three regions reached similar ARR. LA patients had higher baseline rates of comorbidities and incomplete previous remissions than the other two regions, as well as significant improvements in QoL, depression, fatigue, and cognition scores. They also had a better perception of the benefits of a GA conversion in terms of efficacy and AEs than CWE and EE patients.

These discrepancies may be due to differences in healthcare standards and environmental factors between the different regions. For example, the US, Canada, and LA have clinical treatment guidelines that vary in the topics discussed and the use of GA, IFN-β, natalizumab, dalfampridine, and fingolimod [10, 16–18]. Also, it is possible that patients from different regions may have different epidemiological characteristics and comorbidities [16]. These regional differences cannot be adjusted in such a study, where the observational, non-interventional design carries inherent analytical limitations.

Now that consensus guidelines have defined a suboptimal treatment response, and neurodegenerative activity has been identified even in early stages of disease [19, 20], converting to another DMT class represents a logical treatment strategy in patients who fail to respond adequately to first-line treatments. Our results suggest that more attention is required regarding the importance of establishing formal conversion algorithms that account for geographic variability, ensuring that all patients who could benefit from such an approach are managed in a timely and optimal manner.

Despite study limitations, our observations emphasize the importance of changing a therapy regimen, in particular IFN-based, to improve efficacy and/or overcome treatment intolerance that would otherwise compromise compliance. This is in alignment with previous studies of this strategy [9, 21].

## Conclusions

All patients experienced significant improvements in ARR regardless of geographic region. There were differences between regions in patients’ baseline parameters, comorbidities, and reasons for converting to GA treatment. The evolution of guidelines regarding suboptimal treatment response and DMT conversion has the potential to affect strategies for monitoring and treating patients across all geographies and to improve clinical and patient-reported outcomes.

## Additional files

Additional file 1:
**Patient perception of glatiramer acetate treatment efficacy by geographic region.** ^a^ *P* < .0001 for Latin America vs. other geographic areas. (PDF 7 kb) Additional file 2:
**COPITIMIZE: Main Investigator sites.** (DOCX 14 kb)

## Abbreviations

AE: Adverse event
ARD: Annualized rate of deterioration
ARR: Annualized relapse rate
CES-D: Centre for Epidemiologic Studies Depression
CWE: Canada and Western Europe
DMT: Disease-modifying therapy
EDSS: Expanded Disability Status Scale
EE: Eastern Europe
FAMS: Functional Assessment of Multiple Sclerosis
GA: Glatiramer acetate
IFN: Interferon
i.m.: Intramuscular
LA: Latin America
MFIS: Modified Fatigue Impact Scale
MS: Multiple sclerosis
PASAT: Paced Auditory Serial Addition Test
QoL: Quality of life
RMS: Relapsing-remitting multiple sclerosis
s.c.: Subcutaneous
SD: Standard deviation

## References

[1] Aharoni R (2013). The mechanism of action of glatiramer acetate in multiple sclerosis and beyond. Autoimmun Rev.

[2] Koch-Henriksen N, Sørensen PS (2010). The changing demographic pattern of multiple sclerosis epidemiology. Lancet Neurol.

[3] Polman CH, O'Connor PW, Havrdova E, Hutchinson M, Kappos L, Miller DH, Phillips JT, Lublin FD, Giovannoni G, Wajgt A, Toal M, Lynn F, Panzara MA, Sandrock AW, AFFIRM Investigators (2006). A randomized, placebo-controlled trial of natalizumab for relapsing multiple sclerosis. N Engl J Med.

[4] Comi G, Filippi M, Wolinsky JS (2001). European/Canadian multicenter, double-blind, randomized, placebo-controlled study of the effects of glatiramer acetate on magnetic resonance imaging--measured disease activity and burden in patients with relapsing multiple sclerosis. European/Canadian Glatiramer Acetate Study Group. Ann Neurol.

[5] The IFNB, Group MSS (1993). Interferon beta-1b is effective in relapsing-remitting multiple sclerosis. I. Clinical results of a multicenter, randomized, double-blind, placebo-controlled trial. Neurology.

[6] Rudick RA, Stuart WH, Calabresi PA, Confavreux C, Galetta SL, Radue EW, Lublin FD, Weinstock-Guttman B, Wynn DR, Lynn F, Panzara MA, Sandrock AW, SENTINEL Investigators (2006). Natalizumab plus interferon beta-1a for relapsing multiple sclerosis. N Engl J Med.

[7] Coyle PK (2008). Switching algorithms: from one immunomodulatory agent to another. J Neurol.

[8] Goodin DS, Frohman EM, Hurwitz B, O'Connor PW, Oger JJ, Reder AT, Stevens JC (2007). Neutralizing antibodies to interferon beta: assessment of their clinical and radiographic impact: an evidence report: report of the Therapeutics and Technology Assessment Subcommittee of the American Academy of Neurology. Neurology.

[9] Rio J, Tintore M, Sastre-Garriga J, Nos C, Castilló J, Tur C, Comabella M, Montalban X (2012). Change in the clinical activity of multiple sclerosis after treatment switch for suboptimal response. Eur J Neurol.

[10] Freedman MS, Selchen D, Arnold DL, Prat A, Banwell B, Yeung M, Morgenthau D, Lapierre Y, Canadian Multiple Sclerosis Working Group (2013). Treatment optimization in MS: Canadian MS Working Group updated recommendations. Can J Neurol Sci.

[11] Hartung HP, Montalban X, Sorensen PS, Vermersch P, Olsson T (2011). Principles of a new treatment algorithm in multiple sclerosis. Expert Rev Neurother.

[12] Johnson KP (2012). Glatiramer acetate for treatment of relapsing-remitting multiple sclerosis. Expert Rev Neurother.

[13] Scott LJ (2013). Glatiramer acetate: a review of its use in patients with relapsing-remitting multiple sclerosis and in delaying the onset of clinically definite multiple sclerosis. CNS Drugs.

[14] Ziemssen T, Bajenaru OA, Carrá A, de Klippel N, de Sá JC, Edland A, Frederiksen JL, Heinzlef O, Karageorgiou KE, Lander Delgado RH, Landtblom AM, Macías Islas MA, Tubridy N, Gilgun-Sherki Y (2014). A 2-year observational study of patients with relapsing-remitting multiple sclerosis converting to glatiramer acetate from other disease-modifying therapies: the COPTIMIZE trial. J Neurol.

[15] Kulesher R, Forrestal E (2014). International models of health systems financing. J Hosp Admin.

[16] Carrá A, Macias-Islas MA, Gabbai AA, Correale J, Bolaña C, Sotelo ED, Bonitto JG, Vergara-Edwards F, Vizcarra-Escobar D (2011). Optimizing outcomes in multiple sclerosis: consensus guidelines for the diagnosis and treatment of multiple sclerosis in Latin America. Ther Adv Neurol Disord.

[17] Finkelsztejn A, Fragoso YD, Ferreira ML, Lana-Peixoto MA, Alves-Leon SV, Gomes S, Damasceno BP, Mendes MF, Salgado PR, Correa EC, Comini-Frota ER, Diniz DS, Gama PD, Kaimen-Maciel DR, Morales RR, Arruda WO, Grzesiuk AK, Khouri JM, Lopes JS, Rocha CF, Domingues R, Gonçalves MV, Lorenti MA, Parolin MK, Siquineli F, Tosta ED, Brooks JB, Gallina AS, Melges LD, Ruocco HH (2011). The Brazilian database on pregnancy in multiple sclerosis. Clin Neurol Neurosurg.

[18] Miller RM, Happe LE, Meyer KL, Spear RJ (2012). Approaches to the management of agents used for the treatment of multiple sclerosis: consensus statements from a panel of U.S. managed care pharmacists and physicians. J Manag Care Pharm.

[19] Filippi M, Bozzali M, Rovaris M, Gonen O, Kesavadas C, Ghezzi A, Martinelli V, Grossman RI, Scotti G, Comi G, Falini A (2003). Evidence for widespread axonal damage at the earliest clinical stage of multiple sclerosis. Brain.

[20] De Stefano N, Narayanan S, Francis GS, Arnaoutelis R, Tartaglia MC, Antel JP, Matthews PM, Arnold DL (2001). Evidence of axonal damage in the early stages of multiple sclerosis and its relevance to disability. Arch Neurol.

[21] Gajofatto A, Bacchetti P, Grimes B, High A, Waubant E (2009). Switching first-line disease-modifying therapy after failure: impact on the course of relapsing-remitting multiple sclerosis. Mult Scler.

