# Supplementary figures and images for: Sub-analysis of geographical variations in the 2-year observational COPTIMIZE trial of patients with relapsing–remitting multiple sclerosis converting to glatiramer acetate
Source: BMC Neurol. 2015 Oct 8;15:189. doi: 10.1186/s12883-015-0448-4 (PMC4599648; doi:10.1186/s12883-015-0448-4)

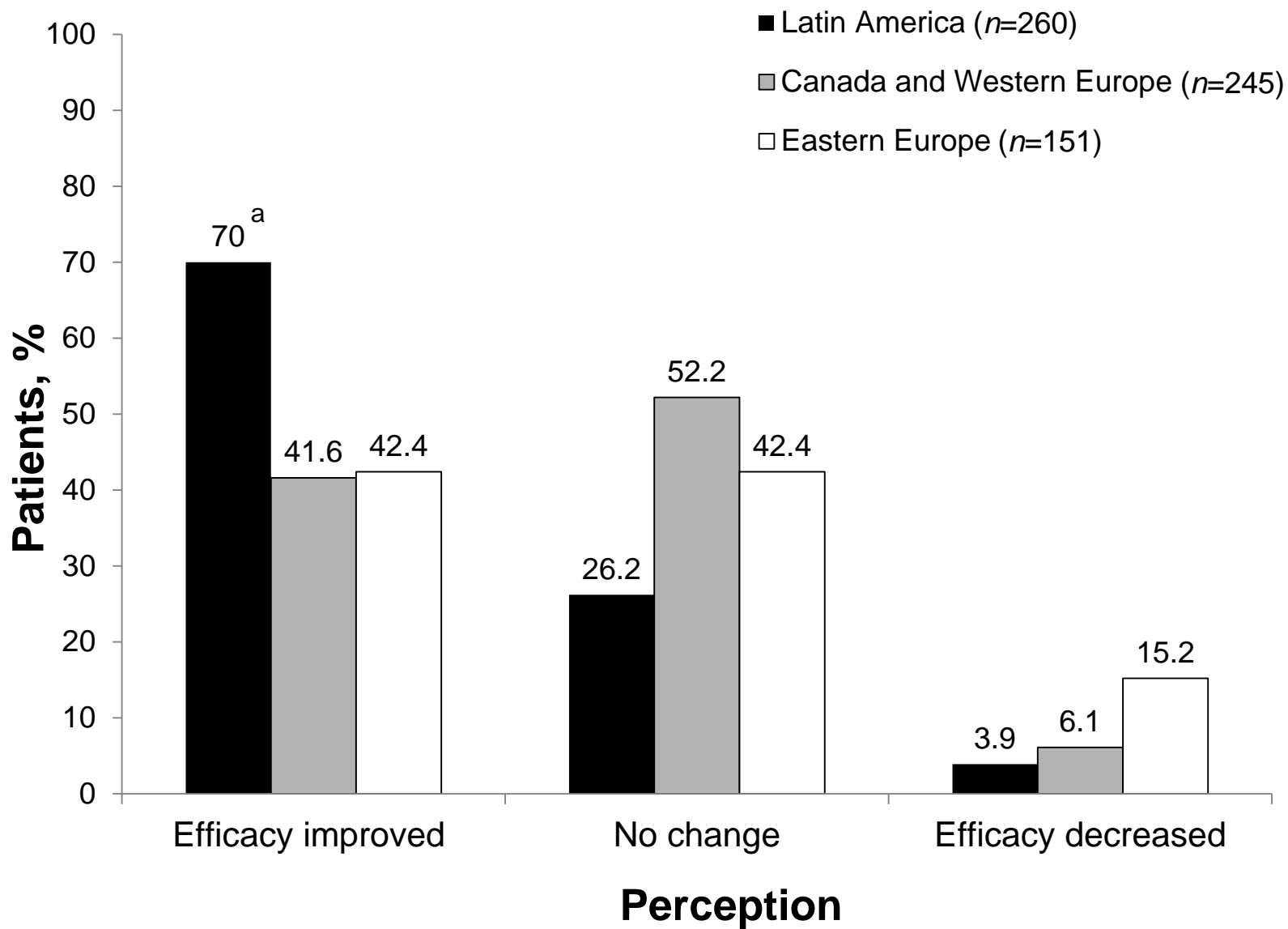

Supplement: Additional file 1: — Patient perception of glatiramer acetate treatment efficacy by geographic region. a P < .0001 for Latin America vs. other geographic areas. (PDF 7 kb) [file 12883_2015_448_MOESM1_ESM.pdf]
