# Supplementary material for: Sub-analysis of geographical variations in the 2-year observational COPTIMIZE trial of patients with relapsing–remitting multiple sclerosis converting to glatiramer acetate
Source: BMC Neurol. 2015 Oct 8;15:189. doi: 10.1186/s12883-015-0448-4 (PMC4599648; doi:10.1186/s12883-015-0448-4)
Supplement: Additional file 2: — COPITIMIZE: Main Investigator sites. (DOCX 14 kb) [file 12883_2015_448_MOESM2_ESM.docx]

**COPITIMIZE: Main Investigator sites**

| **Country** | **Institute** |
| --- | --- |
| Argentina | Hospital Británico de Buenos Aires |
| Belgium | Jessa Ziekenhuis-Campus Virga Jesse |
| Brazil | Universidade de São Paulo |
| Canada | Burnaby Hospital |
| Chile* |  |
| Denmark | Glostrup Hospital |
| France | Poissy Hospital |
| Germany | Neurologische Universitätsklinik,  Klinikum Carl Gustav Carus |
| Greece | General National Hospital of Athens |
| Hungary | Peterfy Hospital Budapes |
| Ireland | St Vincent’s University Hospital |
| Mexico | University of Guadalajara |
| The Netherlands* |  |
| Norway | Sykehuset Buskerud HF |
| Portugal | Hospital de Santa Maria |
| Romania | Spitalul Universitar de Urgenta Bucuresti |
| Slovakia | SM Center Bratislava |
| Sweden | Motala Hospital |
| Venezuela | Centro Médico Docente La Trinidad  Centro Médico de Caracas |
| Taiwan | Chang Gung Memorial Hospital, Taipei, Taiwan |

*No local requirement for IRB approval
